# Characterization of Digestive Involvement in Patients with Chronic *T. cruzi* Infection in Barcelona, Spain

**DOI:** 10.1371/journal.pntd.0003105

**Published:** 2014-08-21

**Authors:** María-Jesús Pinazo, Gloria Lacima, José-Ignacio Elizalde, Elizabeth-Jesús Posada, Fausto Gimeno, Edelweiss Aldasoro, María-Eugenia Valls, Joaquim Gascon

**Affiliations:** 1 Barcelona Centre for International Health Research (CRESIB), Hospital Clínic i Provincial/IDIBAPS, Universitat de Barcelona, Barcelona, Spain; 2 Gastrointestinal Motility Unit, Gastroenterology Department, Hospital Clinic, Barcelona, Spain; 3 Gastroenterology Department, Hospital Clínic, Barcelona, Spain; 4 Radiology Department, CDIC, Hospital Clínic, Barcelona, Spain; 5 Microbiology Department, Hospital Clínic, Barcelona, Spain; Emory University, United States of America

## Abstract

**Background:**

Digestive damage due to Chagas disease (CD) occurs in 15–20% of patients diagnosed as a result of peristaltic dysfunction in some endemic areas. The symptoms of chronic digestive CD are non-specific, and there are numerous confounders. Diagnosis of CD may easily be missed if symptoms are not evaluated by a well trained physician. Regular tests, as barium contrast examinations, probably lack the necessary sensitivity to detect early digestive damage.

**Methods:**

71 individuals with *T. cruzi* infection (G1) and 18 without (G2) coming from Latin American countries were analyzed. They were asked for clinical and epidemiological data, changes in dietary habits, and history targeting digestive and cardiac CD symptoms. Serological tests for *T. cruzi*, barium swallow, barium enema, an urea breath test, and esophageal manometry were requested for all patients.

**Principal findings:**

G1 and G2 patients did not show differences in lifestyle and past history. Fifteen (21.1%) of G1 had digestive involvement. Following Rezende criteria, esophagopathy was observed in 8 patients in G1 (11.3%) and in none of those in G2. Manometry disorders were recorded in 34 G1 patients and in six in G2. Isolated hypotensive lower esophageal sphincter (LES) was found in sixteen G1 patients (23.9%) and four G2 patients (28.8%). Achalasia was observed in two G1 patients. Among G1 patients, ineffective esophageal motility was seen in six (five with symptoms), diffuse esophageal spasm in two (one with dysphagia and regurgitation), and nutcracker esophagus in three (all with symptoms). There were six patients with hypertonic upper esophageal sphincter (UES) among G1. Following Ximenes criteria, megacolon was found in ten G1 patients (13.9%), and in none of the G2 patients.

**Conclusions:**

The prevalence of digestive chronic CD in our series was 21.1%. Dysphagia is a non-pathognomonic symptom of CD, but a good marker of early esophageal involvement. Manometry could be a useful diagnostic test in selected cases, mainly in patients with *T. cruzi* infection and dysphagia in whose situation barium swallow does not evidence alterations. Constipation is a common but non-specific symptom that can be easily managed. Testing for CD is mandatory in a patient from Latin America with constipation or dysphagia, and if diagnosis is confirmed, megacolon and esophageal involvement should be investigated.

## Author Summary

Digestive damage due to Chagas disease (CD), which symptoms are non-specific, occurs in 15–20% of patients as a result of peristaltic dysfunction. The results of this study with 71 individuals with *T. cruzi* infection and 18 without it coming from Latin American countries and performed in a non endemic setting showed that the prevalence of digestive chronic CD was 20.8% in *T. cruzi* infected patients. Dysphagia is a non-pathognomonic symptom of CD, but a good marker of early esophageal involvement. Manometry could be a useful diagnostic test in selected cases, mainly in patients with *T. cruzi* infection and dysphagia in whose situation barium swallow does not evidence alterations. Constipation is a common but non-specific symptom that can be easily managed. Testing for CD is mandatory in a patient from Latin America with constipation or dysphagia, and if diagnosis is confirmed, megacolon and esophageal involvement should be investigated.

## Introduction

Due to migratory flows, chronic *Trypanosoma cruzi* infection, or chronic Chagas disease (CD), has become relatively common in traditionally non-endemic countries [1]. This symptomatic form of CD, which affects approximately 40% of patients, is characterized by cardiac and digestive complications that can progress if the disease is not diagnosed and managed early [2], [3]. Approximately 15–20% of patients with chronic CD develop digestive alterations in some endemic areas [3], and although mortality is low, patient quality of life can be severely impaired. Digestive damage due to chronic *T. cruzi* infection occurs as a result of peristaltic dysfunction, and the final stage, megaviscera, is a consequence of neuronal destruction of the enteric nervous system [4], [5], [6]. The damage is variable in terms of progression and extent of involvement of the digestive tract.

The presence of cardiac, digestive, or mixed (cardiac and digestive) involvement has been related to the geographical distribution of *T. cruzi* serotypes [7]–[10]. Cardiac involvement in chronic stages of CD has been widely described [2], [11], but digestive involvement remains poorly characterized in many areas. Digestive megaviscera seems to be more common in central Brazil, less frequent in Bolivia, and practically non-existent in countries north of the Amazon basin, Central America, and Mexico [6]–[8]. Megacolon is usually the final manifestation, since onset of symptoms is slower than in the case of esophageal involvement [12]. In non-endemic areas, the prevalence of digestive manifestations in CD varies according to immigration patterns.

Even if in recent studies [13] dysphagia appeared as a specific and constant symptom in patients with megaesophagus, in general the symptoms of chronic digestive CD are non-specific, and there are numerous confounders, including other common infections [14], [15], megacolon at high altitude, and gastrointestinal problems related to the long-term consumption of coca leaves [16]–[18] or to migration-related changes in diet [19], [20] and stress. A diagnosis of CD may easily be missed if symptoms are not evaluated by a physician who knew of the epidemiology and the symptoms of the disease.

Barium contrast examinations are used to diagnose digestive involvement in CD [21], [22]. and it is possible to establish a classification in four groups based on the severity of the damage following Rezende criteria [22].The value of other diagnostic tools in the detection of early esophageal and colonic involvement in CD patients is unknown.

The objectives of our study were to determine the prevalence of digestive damage in patients with CD at an international health center in a non-endemic area and to study the utility of esophageal manometry in the early diagnosis of esophageal disease.

## Materials and Methods

### Ethics Statement

Written informed consent was obtained from participants before being recruited. Approval for the protocols and for the informed consent was obtained from the Hospital Clínic of Barcelona Ethics Review Committee.

### Design and Setting

This was a prospective study of 89 individuals: 71 with *T. cruzi* infection (Group 1, G1) and 18 without infection but with *T. cruzi*-like digestive symptoms (Group 2, G2). All the patients were from Latin American countries where CD is endemic and were seen at the Center for International Health at a university hospital in Barcelona, Spain.

### Participants and Procedures

Eighty-nine individuals from *T. cruzi*-endemic areas who were over 18 years old and living in Barcelona were invited to participate in the study. After signing the informed consent form, they were asked for clinical and epidemiological data, including age, sex, toxic habits, past medical history, geographical origin, residence in rural environments and mud houses, previous contact with the *T. cruzi* vector, and history of blood donation or transfusion. Changes in dietary habits and weight since arrival in Spain were also recorded. A detailed history targeting digestive and cardiac symptoms of CD was also obtained in each case. Regarding constipation evaluation, Rome III criteria were taken in account [23].

Serological tests for *T. cruzi* and HIV infection and routine blood and biochemical tests (including renal and liver function) were performed in all cases.

Barium swallow, barium enema, a urea breath test, and esophageal manometry were requested for all patients. *T. cruzi*–infected patients were also studied using a protocol that included a 12-lead electrocardiogram, chest X-ray, and echocardiogram. Other tests were performed depending on individuals' symptoms.

Following international recommendations [24], specific treatment with benznidazole (5 mg/kg/d for 60 days) was offered to all patients with *T. cruzi* infection.

### Diagnosis of *T. cruzi* Infection

For the laboratory diagnosis of *T. cruzi* infection, three serum ELISA tests were performed: a commercial ELISA kit with recombinant antigens (BioELISA Chagas, Biokit S.A., Lliçà d'Amunt, Barcelona, Spain), an in-house ELISA kit (whole *T. cruzi* epimastigote antigen) [25], and a conventional ELISA kit (Orthoclinical Diagnostics, Johnson & Johnson Company). Participants were considered to be infected if the results from at least two of the tests were positive [26].

### Radiological Studies

The barium swallow was performed following the Rezende technique [27]. In brief, 200 mL of barium contrast was administered to the patient in an erect position and right anterior oblique projections were taken. The contrast was administered until a column of sufficient height was obtained to ensure passage to the stomach, allowing visualization of the shape, diameter, wall contours, and kinetic activity of the esophagus. X-rays were taken at the time of administration and after 60 seconds.

Barium enema was performed without prior preparation following the Ximenes technique [28]. Briefly, plain abdominal X-rays were obtained and the patient was administered 300 mL of barium contrast diluted in 1200 mL of water to form 1500 mL of suspension, and asked to remain in the right lateral position for 5 minutes. Three radiological projections (dorsal decubitus, ventral decubitus, and lateral) were obtained after contrast administration. Based on the Ximenes criteria, megacolon is diagnosed when the diameter is >6.5 cm in the descending colon, >8 cm in the ascending colon, and >12 cm in the cecum.

### Urea Breath Test

For the urea breath test, which detects *Helicobacter pylori* infection, the patients were administered a capsule containing urea labeled with 13-C, which was then measured in exhaled CO_2_ samples. The principle of the test is based on hydrolysis of the non-radioactive isotope 13-C to CO_2_ by the *H. pylori* enzyme urease. The CO_2_ is then transported by the blood to the lungs, where it is exhaled.

### Esophageal Manometry

Esophageal manometry, also known as an esophageal motility study, was performed in both groups as previously described [29], [30]. Esophageal body motility and lower esophageal sphincter (LES) pressures were measured using a four-lumen radially oriented polyvinyl tube with orifices spaced at 5-cm intervals (98–3113; Mui Scientific Mississauga, Ontario, Canada). Each lumen was perfused with distilled water (0.5 mL/min) using a low compliance hydraulic system (Bios Biotechnology & Software, Barcelona, Spain). Pressures were recorded by means of a pressure transducer (9022K0122; CODAN pvb Medical GmbH, Lensahn, Germany) connected via amplifiers to a chart recorder (Polygraf ID, Medtronic Functional Diagnostics, Skovlunde, Denmark) and analyzed by the Polygram 98 V 2.20 esophageal application (Medtronic Functional Diagnostics). Swallowing was monitored by a sensor wrapped around the patient's neck.

Overnight fasting was required in all cases, and the patients were placed in the supine position following insertion of the manometric tube through the nasal passage. Resting LES pressure was measured with the station pull-through technique, using intragastric pressure as the zero reference. The pull-through was performed three times. LES relaxation was assessed after a minimum of five swallows of 5-ml water and esophageal body peristalsis was assessed after a minimum of ten 5-ml swallows at least 30 seconds apart. We also measured upper esophageal sphincter location and resting pressure and assessed pharyngoesophageal coordination after a minimum of five 5-ml swallows.

### Statistical Analysis

Quantitative variables were presented as medians and interquartile range (IQR). Qualitative variables were reported using absolute frequencies and percentages The analyses were performed using Stata 13 (Stata Corporation, College Station, TX).

## Results

We analyzed 71 individuals with *T. cruzi* infection (G1) and 18 without (G2). The mean age of the patients was 34 (12) years and there were 75 women (84%) (59 in G1 and 16 in G2). The patients were mainly from Bolivia (n = 83, 93%). In G1, 69 patients had at least one digestive symptom and in G2, all the patients were symptomatic. The baseline characteristics of the patients, together with toxic habits, lifestyle, past history, digestive complaints are shown in Table 1, and the results of the urea breath test are shown in Table 2.

**Table 1:** Baseline characteristics of patients, including past gastrointestinal history.

	Group 1 (Chagas +) (N = 71) n (%)	Group 2 (Chagas −) (N = 18) n (%)
**Geographical origin**		
Bolivia	66 (92.9%)	14 (77.8%)
Argentina	3 (4.2%)	2 (11.1%)
Paraguay	2 (2.8%)	0
Colombia	0	2 (11.1%)
**Sex**		
Male	12 (16.9%)	2 (11.1%)
Female	59 (83.1%)	16 (88.9%)
**Age (mean, SD)**	36 (9)	32 (5)
**Lifestyle and toxics habits**		
Tobacco	2 (2.8%)	-
Alcohol intake^1^	15 (21.1%)	5 (27.8%)
Sedentary lifestyle	43 (60.6%)	12 (66.7%)
**Past gastrointestinal history**		
Dyspepsia^2^ or peptic ulcer	6 (8.5%)	5 (27.8%)
Esophageal surgery^3^	1 (1.4%)	-
Colon surgery^4^	5 (7%)	1 (5.6%)
**Frequently used drugs**		
Proton pump inhibitors	8 (11.3%)	1 (5.6%)
Laxatives	1 (1.4%)	-
**Weight changes**	26/41 (63.4%)	2/6 (33%)

1 Alcohol intake was reported as occasional in all cases.

2 Presence of symptoms considered as originating in the gastroduodenal region, in the absence of any organic, systemic, or metabolic disease that is likely to explain the symptoms (Roma III criteria).

3 One patient presented in her past history pyloric stenosis surgery, showing normal esophagogram and barium enema.

4 Five patients presented partial colectomy previously of being admitted in the international health centre. After the surgery, one of them presented megasigma, and another one dolichomegacolon. The other three presented dolichocolon. All of them did not showed alterations in esophagogram.

**Table 2:** Symptoms related to *H. pylori* infection and analysis of symptoms shared with *T. cruzi* esophagopathy.

	Group 1 (Chagas +) n (%)			Group 2 (Chagas −) n (%)	
	UBT +	UBT −	Abnormal BS findings^1^	UBT +	UBT −
**Total cases with UBT + or −**	**54**	**8**	**-**	**12**	**3**
**Dysphagia**	17/54 (31.5%)	5/8 (62.5%)	7/8	1/12 (8.3%)	1/3 (33.3%)
**Heartburn**	18/54 (33.3%)	1/8 (12.5%)	2/8	5/12 (41.7%)	1/3 (33.3%)
**Abdominal bloating**	27/54 (50%)	2/8 (25%)	4/8	8/12 (66.7%)	3/3 (100%)
**Increased thirst**	15/54 (27.8%)	-	3/8	6/12 (50%)	1/3 (33.3%)
**Chest pain**	37/54 (68.5%)	5/8 (62.5%)	6/8	5/12 (41.7%)	2/3 (66.7%)

**Abbreviations: UBT:** urea breath test; **BS:** barium swallow/esophagogram.

1 No abnormal findings in G2.

Based on the criteria of Rezende [27] and Ximenes [28], 15 (21.1%) of the patients with CD in our series had digestive involvement: seven had megacolon, five had esophagopathy, and three had both megacolon and esophagopathy.

### Esophageal Involvement

#### Barium swallow

Following the Rezende criteria [27], esophagopathy (stage I) was observed in 8 patients in G1 (11.3%) and in none of those in G2. The related symptoms are shown in Table 3.

**Table 3:** Signs and symptoms of esophagopathy and association with esophagogram findings.

	Group 1 (*T. cruzi-*positive) (N = 71)	
	Patients with clinical symptoms n (%)	Esophagopathy (Stage I) (n)
**Symptoms** 1		
Dysphagia S	6/71 (8.4)	1
Dysphagia L	11/71(15.5)	3
Dysphagia L and S	8/71 (11.3)	3
Odynophagia	4/71 (5.6)	2
Regurgitation	16/71 (22.5)	3
Chest pain	41/71 (57.7)	6

**Abbreviations: LES:** lower esophageal sphincter; **L:** in relation to liquids; **S:** in relation to solids.

1 More than one symptom in some patients.

#### Manometry

Esophageal manometry was performed in 67 patients in G1 and in 14 in G2. Manometry disorders were recorded in 34 patients in the first group (51.5%) and in six (42.8%) in the second. Sixteen G1 patients (23.88%) and four G2 patients (28.76%) had isolated hypotensive LES. Achalasia was observed in two patients, both from G1. Also in G1, ineffective esophageal motility was seen in six patients (five with symptoms), diffuse esophageal spasm in two (one of whom had dysphagia and regurgitation), and nutcracker esophagus in three (all with symptoms).

Only one G2 patient with isolated hypotensive LES showed hypertonic upper esophageal sphincter (UES). In G1, there were six patients with hypertonic UES (one without symptoms, four with symptoms but without LES involvement, and one with symptoms and ineffective esophageal motility and hypotonic LES).

The manometry results shown by symptoms for both groups are summarized in Table 4.

**Table 4:** Manometric patterns and association with symptoms.

	Group 1 (*T. cruzi*-positive) (N = 67) n (%)					Group 2 (*T. cruzi-*negative) (N = 14) n (%)
Manometric Patterns	All	Asymptomatic	Symptomatic			
			Dysphagia	Chest pain	Regurgitation	
**Normal**	33(48.5)		11/33 (33)	23/33 (70)	4/33(12)	8 (57.1)
**Achalasia**	2 (2.9)	0	2/2 (100)	1/2 (50)	2/2 (100)	0
**Atypical disorders of LES relaxation**	1(1.5)	0	1/1 (100)	0	0/1	0
Diffuse esophageal spasm	2 (2.9)	0	1/2(50)	0	1/2(50)	0
Nutcracker esophagus	3 (4.4)	0	1/3(33)	2/3 (66)	1/3(33)	0
Ineffective esophageal motility	6 (8.8)	0	3/6 (50)	5/6 (8)	0/6	0
Isolated hypotensive LES	16 (23.5)	3	4/16 (25)	11/16 (69)	1/16 (6)	4 (28.6)
**Non-specific esophageal motility abnormalities**	4 (5.6)	0	2/4 (50)	3/4 (75)	2/4 (50)	2 (14.3)

**Abbreviation: LES:** lower esophageal sphincter.

### Colonic Involvement: Barium Enema

Megacolon was found in 10 G1 patients (13.9%), three of whom had stage I esophagopathy, and in none of the G2 patients. Additionally, dolichocolon (colonic elongation that exceeds the pelvis) was observed in 44 patients in G1 (62%) and in 14 patients (77.8%) in G2. A summary of colonopathy-related signs and symptoms is shown in Table 5.

**Table 5:** Colonopathy: Clinical findings and barium enema results.

	Group 1 (N = 71)			Group 2 (N = 18)		
	Patients with clinical symptoms n (%)	Dolichocolon n (%)	Megacolon n (%)	Patients with clinical symptoms n (%)	Dolichocolon n (%)	Megacolon n (%)
**Patients diagnosed by barium enema**	-	44 (62)	10 (14)	-	14 (77.8)	-
**Signs/symptoms**						
Diffuse abdominal pain	7 (9.8)	4/44 (9.1)	1/10 (10)	-	-	-
Constipation	48 (67.6)	32/44 (72.7)	8/10 (80)	16/18 (88.9)	13/14 (92.9)	-

Regarding Rome III criteria, constipation was found in 48 G1 patients, and among them 12 presented one deposition every 48 hours, 11 every 48–72 hours and 17 every 72 or more hours. The eight that with constipation that did not presented constipation presented other constipation defining symptoms.

## Discussion

While cardiac involvement has been well described in CD, gastrointestinal manifestations have received considerably less attention. Digestive involvement in chronic CD has only been characterized in certain regions of Brazil, with reports dating back to the 1950s [12], [31]–[33]. Additionally, the techniques used to diagnose megaesophagus [27] and megacolon [28] have not been updated since they were first introduced some 60 years ago. We have presented the results of digestive damage in a series of Latin American patients with and without *T. cruzi* infection seen at an international health center in Barcelona, Spain. The patients were mainly from Bolivia, and there are few reports describing the characteristics of CD in this population [34] Furthermore, few studies have been performed in Bolivia.

Approximately one in five patients with CD in our series had digestive problems, a rate that is similar to figures reported in other series [3], [34]. Their symptoms were similar to those observed in patients without *T. cruzi* infection from the same geographical area. Due to the non-specificity of gastrointestinal manifestations in chronic CD, suspicion of *T. cruzi* infection is key to making a diagnosis and serology is mandatory for a definitive diagnosis and treatment. Regarding specific treatment, it was offered to all patients that fulfill criteria (64 among 71), and all patients accepted and completed it.

Eight patients among G1 showed stage I esophagopathy in the barium swallow, six women and two men. The prevalence of megaesophagus by gender has resulted in many studies with controversial results. In a recent study [35] a comparative analyses between men and women showed that women presented higher prevalence of stage I involvement. In our series, there is a clear predominance of young women: even if our results are similar to those already published, we cannot conclude that it was a statistically significant difference due to the gender bias of our cohort. On the other hand, the mean age of Salibaet al. cohort was 54.4 years, and a significant increase in age was observed in advanced megaesophagus groups. In our series, the mean age was lower, so that could explain the less esophagopathy progression showed by patients.

Dysphagia is a major symptom of esophageal involvement in CD. In our series, it was detected in seven of the eight patients diagnosed with stage I esophagopathy, but it was also seen in 15 other patients in G1 who had normal barium swallow and manometry findings.

Hypertonic LES, a criterion of Chagasic esophagopathy, [32], [33], [35], [36] was found in three patients in G1, all of whom had dysphagia. No cases were seen in G2. Hypotonic LES was observed in 16 (23%) of the 67 patients who underwent manometry in G1 and in four (28.6%) of the 14 patients in G2, indicating that hypotonic LES is probably related to gastroesophageal reflux disease and not to CD. Atypical chest pain was reported by 41 G1 patients (57.7%) and eight G2 patients (44.4%). Of the patients with atypical chest pain, four had hypotensive LES on manometry and in G1, the patients had abnormal barium swallow and manometric findings. Atypical chest pain is a non-specific symptom that can be related to heart damage or other conditions, but it has occasionally been linked to impaired esophageal microcirculation and has also been described as a means of expressing pain due to swallowing in patients with CD [3]

Achalasia diagnosed by manometry was observed in two G1 patients, both of whom had dysphagia and regurgitation and stage I esophagopathy in the barium swallow. The three patients with nutcracker oesophagus in G1 were all symptomatic but had normal barium swallow findings. On the other hand, studies in Central Brazil shows that around 10% of achalasia was idiopathic, and even if patients came for CD endemic regions, in some countries of Europe the incidence of new cases of achalasia ranged between 0.018/100.000/year [37] and 1.59 cases/100.000/year [38], [39].

It is important to highlight the correlation between manometric and barium swallow and clinical findings. The eight patients with stage I esophagopathy were symptomatic and six had abnormal manometric findings. Furthermore, 26 of the 33 patients with normal manometric findings had a normal esophagram. In G1, 10 of the 26 patients with a normal esophagogram had clearly detectable alterations in manometry: atypical LES relaxation in one patient with dysphagia, diffuse esophageal spasm in one patient with regurgitation, nutcracker oesophagus in three patients (one with dysphagia, one with chest pain, and one with regurgitation), and ineffective esophageal motility in five patients (two with dysphagia, two with chest pain, and one with no symptoms). While manometry has been proposed as a new technique with diagnostic value in early esophageal damage due to CD, it is a time-consuming procedure that, furthermore, is unavailable in most health care centers. Our findings indicate, however, that it could be useful for detecting incipient esophageal alterations in asymptomatic patients with *T. cruzi* infection and normal barium swallow findings.

Constipation was a common symptom in the two groups, and was observed in eight of the 10 patients with megacolon. Dolichocolon, a sedentary lifestyle, and changes in dietary habits could be at the root of this symptom [34]. Even though constipation is non-specific, it should not be overlooked in *T. cruzi* infection because of its potential clinical implications. In all the cases in our series, constipation was adequately controlled with symptomatic treatment, including dietary changes and, in some cases, laxatives. None of the patients with megacolon required surgical treatment.

Dolichocolon has not traditionally been considered a sign of CD, and the results of our study confirm that it probably is not. Dolichocolon can also be caused by embryological elongation of the sigmoid, and in Bolivian patients, it could be related to other factors such as altitude or long-term consumption of coca leaves [17].

The prevalence of *H. pylori* infection was high in our series, consistent with previous reports [15]. Some authors have hypothesized on a potential relationship between *T. cruzi* and *H. pylori* infections [14], probably in relation to common environmental risk factors. However, it has been suggested that hypochlorhydria and/or other gastric dysfunctions caused by *T. cruzi* infection could facilitate *H. pylori* transmission [14].

Classic *H. pylori* infection symptoms (abdominal bloating, heartburn, and increased thirst) were common in the two groups, and are probable confounding factors in the evaluation of patients with CD. In any case, co-infections should be diagnosed and treated in order to ameliorate the symptoms.

One limitation of our study is that it was designed and started before high-resolution manometry was available at our center. It is likely that esophageal pressure topography analysis would have detected more esophageal motility disorders than conventional manometry. However, to guarantee uniformity and taking into account that high-resolution manometry is not yet widely available, all studies were performed using conventional manometry.

### Conclusions

The prevalence of digestive chronic CD in our series (21.1%) was at the higher end of the estimated prevalence range. Dysphagia is a non-pathognomonic symptom of CD, but a good marker of esophageal involvement, even in early stages of the disease. Manometry could be a useful diagnostic test in selected cases, mainly in patients with *T. cruzi* infection and dysphagia in whom barium swallow does not evidence alterations. *H. pylori* co-infection was common in this series of mostly Bolivian patients, and the management of this condition may contribute to relieving digestive symptoms in patients with chronic CD.

Constipation is a common but non-specific symptom that can cause significant discomfort but is easily managed. Testing for CD is mandatory in a patient from Latin America with constipation or dysphagia, and if diagnosis is confirmed, megacolon and esophageal involvement should be investigated.

## Supporting Information

Checklist S1 STROBE checklist.
(DOC)

## References

[1] Gascon J, Bern C, Pinazo MJ (2010) Chagas disease in Spain, the United States and other non-endemic countries. Acta Trop 115: 22–27. 19646412 10.1016/j.actatropica.2009.07.019

[2] Kirchhoff LV, Weiss LM, Wittner M, Tanowitz HB (2004) Parasitic diseases of the heart. Front Biosc 9: 706–723. 10.2741/1255 14766402

[3] Prata A (2001) Clinical and epidemiological aspects of Chagas disease. Lancet Infect Dis 1: 92–100. 11871482 10.1016/S1473-3099(01)00065-2

[4] Köberle F (1968) Chagas' disease and Chagas' syndromes: the pathology of American trypanosomiasis. Adv Parasitol 6: 63–116. 4239747 10.1016/s0065-308x(08)60472-8

[5] Iantorno G, Bassotti G, Kogan Z, Lumi CM, Cabanne AM, et al (2007) The enteric nervous system in chagasic and idiopathic megacolon. Am J Surg Pathol 31: 460–468. 17325489 10.1097/01.pas.0000213371.79300.a8

[6] Meneghelli UG (2004) Chagasic enteropathy. Rev Soc Bras Med Trop 37: 252–260. 15330067 10.1590/s0037-86822004000300012

[7] Andrade SG, Magalhaes JB (1996) Biodemes and zymodemes of *Trypanosoma cruzi* strains: correlations with clinical data and experimental pathology. Rev Soc Bras Med Trop 30: 27–35. 10.1590/s0037-86821997000100006 8993106

[8] Anez N, Crisante G, da Silva FM, Rojas A, Carrasco H, et al (2004) Predominance of lineage I among *Trypanosoma cruzi* isolates from Venezuelan patients with different clinical profiles of acute Chagas' disease. Trop Med Int Health 9: 1319–1326. 15598264 10.1111/j.1365-3156.2004.01333.x

[9] Burgos JM, Begher SB, Freitas JM, Bisio M, Duffy T, et al (2005) Molecular diagnosis and typing of *Trypanosoma cruzi* populations and lineages in cerebral Chagas disease in a patient with AIDS. Am J Trop Med Hyg 73: 1016–1018. 16354804

[10] Burgos JM, Diez M, Vigliano C, Bisio M, Risso M, et al (2010) Molecular identification of *Trypanosoma cruzi* discrete typing units in end-stage chronic Chagas heart disease and reactivation after heart transplantation. Clin Infect Dis 51: 485–495. 20645859 10.1086/655680

[11] Punukollu G, Gowda RM, Khan IA (2004) Early twentieth century descriptions of the Chagas heart disease. Int J Cardiol 95: 347–349. 15193845 10.1016/j.ijcard.2003.04.046

[12] Rezende JM, Moreira H (2000) Forma digestiva da Doença de Chagas. In: Brener Z AZ, Barral-Neto N, editor. *Trypanosoma cruzi* e Doeça de Chagas. Rio de Janeiro: Guanabara Koogan. pp. 297–343.

[13] Souza DH, Vaz M da G, Fonseca CR, Luquetti A, Rezende Filho J, et al (2013) Current epidemiological profile of Chagasic megaesophagus in Central Brazil. Rev Soc Bras Med Trop 46 3: 316–321. 23856859 10.1590/0037-8682-0065-2013

[14] Nascimento RS, Valente SR, Oliveira LC (2002) Seroprevalence of Helicobacter pylori infection in chronic chagasic patients, and in the rural and urban population from Uberlandia, Minas Gerais, Brazil. Rev Inst Med Trop Sao Paulo 44: 251–254. 12436163 10.1590/s0036-46652002000500003

[15] Pinazo MJ, Ignacio Elizalde J, de Jesus Posada E, Gascon J (2010) Co-infection with two emergent old pathogens: *Trypanosoma cruzi* and *Helicobacter pylori* . Enferm Infecc Microbiol Clin 28: 751–752. 20580134 10.1016/j.eimc.2010.03.014

[16] Anand AC, Sashindran VK, Mohan L (2006) Gastrointestinal problems at high altitude. Trop Gastroenter 27: 147–153. 17542291

[17] Frisancho O (2008) Dolichomegacolon of the Andes and intestinal volvulus due to altitude. Rev Gastroenterol Peru 28: 248–257. 18958141

[18] Patino F, Ponce R, Lora J, Aguilar C, Rios Dalenz J (1983) Megacolon at high altitude. Acta Gastroenterol Latinoam 13: 699–703. 6680261

[19] Geraix J, Ardisson LP, Marcondes-Machado J, Pereira PC (2007) Clinical and nutritional profile of individuals with Chagas disease. Braz J Infect Dis 11: 411–414. 17873995 10.1590/s1413-86702007000400008

[20] Posada E PC, Angulo N, Pinazo MJ, Gimeno F, Elizalde I, et al (2011) Bolivian migrants with Chagas disease in Barcelona, Spain: a qualitative study of dietary changes and digestive problems. International Health 3: 289–294. 24038501 10.1016/j.inhe.2011.09.005

[21] Kamiji MM, de Oliveira RB (2005) Features of Chagas' disease patients with emphasis on digestive form, in a tertiary hospital of Ribeirao Preto, SP. Rev Soc Bras Med Trop 38: 305–309. 16082476 10.1590/s0037-86822005000400005

[22] Rezende JM (2006) Diagnóstico das manifestações digestivas da Doença de Chagas. Enfermedades emergentes 9: 37–39.

[23] Drossman DA, Dumitrascu DL (2006) Rome III: New standard for functional gastrointestinal disorders. J Gastrointestin Liver Dis 15 3: 237–241. 17013448

[24] Bern C, Montgomery SP, Herwaldt BL, Rassi A Jr, Marin-Neto JA, et al (2007) Evaluation and treatment of Chagas disease in the United States: a systematic review. JAMA 298: 2171–2181. 18000201 10.1001/jama.298.18.2171

[25] Riera C, Vergés M, López-Chejade P, Piron M, Gascon J, et al (2009) Desarrollo y evaluación de una técnica ELISA con antígeno crudo de *Trypanosoma cruzi* para el diagnóstico de la enfermedad de Chagas. Enfermedades emergentes 11: 22–19.

[26] WHO (2002) WHO Technical Report Series Nr. 905, Control of Chagas disease, 2^nd^ report 2002, 24–28. 12092045

[27] de Rezende J, Lauar KM, de OA (1960) Clinical and radiological aspects of aperistalsis of the esophagus. Rev Bras Gastroenterol 12: 247–262. 13741121

[28] Ximenes CARJ, Moreira H, Vaz MG (1984) Técnica simplificada para o diagnóstico radiológico do Megacolon chagásico. Rev Soc Bras Med Trop 17–23.

[29] Grande L, Lacima G, Ros E, Pera M, Ascaso C, et al (1999) Deterioration of esophageal motility with age: a manometric study of 79 healthy subjects. Am J Gastroenterol 94: 1795–1801. 10406237 10.1111/j.1572-0241.1999.01208.x

[30] Grande L LGSGftSoDM (1998) Study of esophageal function by standard esophageal manometry in 72 healthy volunteers. Proposal for national reference values. Rev Esp Enferm Dig 90: 613–618. 9780798

[31] Pera JS (1953) Chagas heart disease and megacolon and megaesophagus. Rev Bras Med 10: 515–517. 13112648

[32] Prata A (1960) Etiological relationship between Chagas' disease and megaesophagus. Revista brasileira de medicina 17: 300–308. 13737750

[33] de Rezende JM (1969) Chagas' disease of the gastrointestinal tract: megaesophagus, megacolon and denervation. Med Monatsschr 23: 344–350. 4981863

[34] Perez-Ayala A, Perez-Molina JA, Norman F, Monge-Maillo B, Faro MV, et al (2011) Gastro-intestinal Chagas disease in migrants to Spain: prevalence and methods for early diagnosis. Ann Trop Med Parasitol 105: 25–29. 21294946 10.1179/136485910X12851868780423 PMC4089794

[35] Moraes-Filho JP, Bettarello A (1989) Response of the lower esophageal sphincter to pentagastrin on patients with megaesophagus secondary to Chagas' disease. Rev Hosp Clin Fac Med Sao Paulo 44: 178–180. 2517711

[36] Silva LC, Vicentine FP, Herbella FA (2012) High resolution manometric findings inpatients with Chagas' disease esophagopathy. Asian Pac J Trop Med 5: 110–112. 22221752 10.1016/S1995-7645(12)60006-6

[37] Marlais M, Fishman JR, Fell JM, Haddad MJ, Rawat DJ (2011) UK incidence of achalasia: an 11-year national epidemiological study. Arch Dis Child 96 2: 192–194. 20515971 10.1136/adc.2009.171975

[38] Gennaro N, Portale G, Gallo C, Rocchietto S, Caruso V, et al (2011) Esophageal achalasia in the Veneto region: epidemiology and treatment. Epidemiology and treatment of achalasia. J Gastrointest Surg 15 3: 423–428. 21116729 10.1007/s11605-010-1392-7

[39] Birgisson S, Richter JE (2007) Achalasia in Iceland, 1952–2002: an epidemiologic study. Dig Dis Sci 52 8: 1855–1860. 17420933 10.1007/s10620-006-9286-y

